# New insights into the central sympathetic hyperactivity post‐myocardial infarction: Roles of METTL3‐mediated m^6^A methylation

**DOI:** 10.1111/jcmm.17183

**Published:** 2022-01-17

**Authors:** Lei Qi, Hui Hu, Ye Wang, Hesheng Hu, Kang Wang, Pingjiang Li, Jie Yin, Yugen Shi, Yu Wang, Yuepeng Zhao, Hangji Lyu, Meng Feng, Mei Xue, Xinran Li, Yan Li, Suhua Yan

**Affiliations:** ^1^ Department of Cardiology The First Affiliated Hospital of Shandong First Medical University & Shandong Provincial Qianfoshan Hospital Shandong Medicine and Health Key Laboratory of Cardiac Electrophysiology and Arrhythmia Jinan China; ^2^ Shandong First Medical University & Shandong Academy of Medical Sciences Jinan China; ^3^ Department of Cardiology Jining No.1 People' Hospital Jining China; ^4^ Department of Cardiology Shandong Qianfoshan Hospital Cheeloo College of Medicine Shandong University Jinan China; ^5^ Medical Research Center The First Affiliated Hospital of Shandong First Medical University & Shandong Provincial Qianfoshan Hospital Shandong Medicine and Health Key Laboratory of Cardiac Electrophysiology and Arrhythmia Jinan China

**Keywords:** m^6^A, METTL3, microglia, myocardial infarction, paraventricular nucleus, sympathetic hyperactivity, TLR4/NF‐κB

## Abstract

Ventricular arrhythmias (VAs) triggers by sympathetic nerve hyperactivity contribute to sudden cardiac death in myocardial infarction (MI) patients. Microglia‐mediated inflammation in the paraventricular nucleus (PVN) is involved in sympathetic hyperactivity after MI. N6‐methyladenosine (m^6^A), the most prevalent mRNA and epigenetic modification, is critical for mediating cell inflammation. We aimed to explore whether METTL3‐mediated m^6^A modification is involved in microglia‐mediated sympathetic hyperactivity after MI in the PVN. MI model was established by left coronary artery ligation. METTL3‐mediated m^6^A modification was markedly increased in the PVN at 3 days after MI, and METTL3 was primarily located in microglia by immunofluorescence. RNA‐seq, MeRIP‐seq, MeRIP‐qPCR, immunohistochemistry, ELISA, heart rate variability measurements, renal sympathetic nerve activity recording and programmed electrical stimulation confirmed that the elevated toll‐like receptor 4 (TLR4) expression by m^6^A modification on TLR4 mRNA 3'‐UTR region combined with activated NF‐κB signalling led to the overwhelming production of pro‐inflammatory cytokines IL‐1β and TNF‐α in the PVN, thus inducing the sympathetic hyperactivity and increasing the incidence of VAs post‐MI. Targeting METTL3 attenuated the inflammatory response and sympathetic hyperactivity and reduced the incidence of VAs post‐MI.

## 1 INTRODUCTION

Sudden cardiac death (SCD) continues to be a major public health challenge, accounting for approximately 20% of all mortality in industrialized countries,^1^ while ventricular fibrillation (VF) is considered the final underlying mechanism of deaths.^2^, ^3^, ^4^ In myocardial infarction (MI) patients,^5^, ^6^ ventricular arrhythmias (VAs), primarily ventricular tachycardia (VT)/VF, are the leading causes of SCD. Driven by this high‐societal impact, profound investigation and understanding of the pathophysiology of ischaemia‐induced VAs has attracted scientific efforts, aiming to further reduce the incidence of SCD.

Sympathetic activation, been known for decades,^7^ plays a vital role in the pathogenesis of VAs during acute MI (AMI) by overwhelming local norepinephrine (NE) release, which contributes to the high mortality by lowering ventricular fibrillation thresholds in AMI^8^, ^9^, ^10^, ^11^ and is highly arrhythmogenic in the peri‐infarct area.^12^, ^13^ Such local NE release from sympathetic nerve terminals is always elicited by central activation following ischaemia^13^ by stimulating efferent myocardial nerve fibers.^12^, ^14^ The paraventricular nucleus (PVN), the key and advanced cardiovascular regulatory region of the brain,^15^, ^16^ is located in the hypothalamus of the central nervous system (CNS), receives and integrates incoming cardiac reflex information and regulates peripheral sympathetic activities via feedback of related neural signalling pathways.^17^ Recent studies by our group and others have shown that microglia‐mediated inflammation in the PVN stimulates sympathetic activation, thus leading to myocardial intrinsic sympathetic hyperactivity after MI.^11^, ^18^, ^19^, ^20^ We have previously demonstrated that toll‐like receptor 4 (TLR4) is highly expressed in microglia and induces sympathetic hyperactivity through the NF‐κB signalling pathway post‐MI.^21^ However, little is known about the precise mechanisms of the post‐transcriptional regulation of TLR4 expression regulation in the PVN post‐MI.

N6‐methyladenosine (m^6^A) is the most abundant mRNA and epigenetic modification^22^, ^23^ that occurs at the post‐transcriptional level in mammals. m^6^A is installed onto target mRNA through m^6^A regulators which include the m^6^A methyltransferase complex (METTL3, METTL14, WTAP, VIRMA, RBM15 and ZC3H13)^24^ and is enriched near the stop codon, 3′‐untranslated region (3′UTR)^25^ and long internal exon, primarily occurring in the RRACH sequence (where R = G or A; H = A, C or U) of mRNA. m^6^A was removed by demethylase (FTO and ALKBH5).^26^ Emerging evidence has shown the biological and pathophysiological significance of m^6^A in human physiology and cancers by endowing controllable protein production^22^, ^27^, ^28^, ^29^ and mRNA stabilization,^30^ and by regulating RNA degradation, splicing, output and translation.^31^ Recently, studies have reported the key role of m^6^A in mediating the inflammatory response in vitro and in vivo. For instance, METTL3 inhibited inflammation in response to lipopolysaccharides (LPS) by MAPK and NF‐κB pathways in human dental pulp cells (HDPCs)^32^; m^6^A attenuated inflammation of rheumatoid arthritis (RA) through the NF‐κB signalling pathway.^33^ However, whether m^6^A is involved in the inflammatory response and regulates the TLR4/NF‐κB signalling pathway in the PVN post‐MI is unknown so far.

This study demonstrated the pathophysiological roles of m^6^A methyltransferases, METTL3, involved in central sympathetic activation via promoting m^6^A installation post‐MI. The molecular mechanism of m^6^A modification mediated by METTL3 was explored by identifying downstream target genes and signalling pathways. Resultingly, the METTL3 gene in the PVN was knocked down by infection with adeno‐associated virus (AAV)‐mediated short hairpin RNA (shRNA). The effects of METTL3‐mediated m^6^A modification on sympathetic activation were investigated. The results of this study further elucidate the related pathological processes and provide a novel therapeutic target for reducing VAs and SCD post‐MI.

## 2 MATERIALS AND METHODS

### 2.1 Animal and MI model

Male Sprague‐Dawley rats (8 weeks old, 260–280 g, Vital River Company; Beijing, China) were housed under a 12 h light/dark at 22–24°C with free access to water and food. This study was conducted in accordance with the guidelines approved by the Committee on Animal Care and Use of the First Affiliated Hospital of Shandong First Medical University (Protocol number: S 030).

**FIGURE 1:**
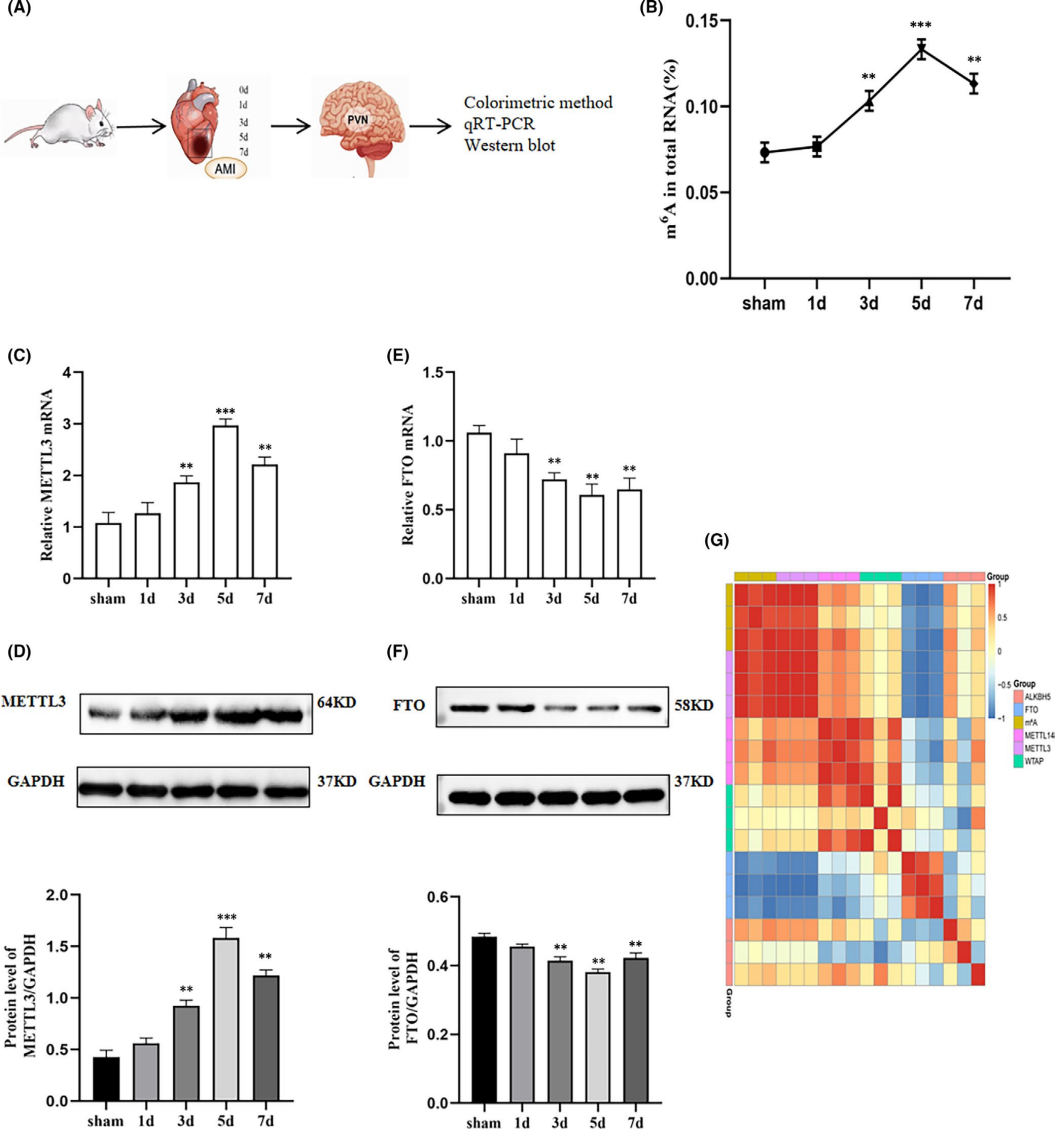
Total m^6^A level and expression of m^6^A enzymes in the PVN of sham and MI groups. (A) Diagram of the experiment. (B) Quantification of total m^6^A was determined by an antibody‐based colorimetric method in the PVN of the sham group and MI groups (at 1, 3, 5 and 7 days). m^6^A enzymes METTL3 was analysed at mRNA (C) and protein (D) levels in the PVN of the sham group and MI groups (at 1, 3, 5 and 7 days) by quantitative real‐time PCR and Western blot analysis. m^6^A enzymes FTO was analysed at mRNA (E) and protein (F) levels in the PVN of the sham group and MI groups (at 1, 3, 5 and 7 days) by quantitative real‐time PCR and Western blot analysis. GAPDH served as an internal control. (G) Heatmap of Pearson correlation analysis between total m^6^A levels and m^6^A‐related enzymes using qRT‐PCR data. Red and blue indicate the positive and negative correlations, respectively. The plots were plotted in the R package heatmap. *n* = 9 per group. Data are presented as mean ± *SD*. **p < 0.01 and ***p < 0.001 versus sham group. Abbreviations: MI, myocardial infarction; PVN, paraventricular nucleus; SD, standard deviation

The rat MI model was established as previously described,^34^ with prior anaesthesia by intraperitoneal injection of 3% sodium pentobarbital (30 mg/kg). A fully anaesthetized state (no response to toe pinching) was confirmed, a tracheotomy was performed, and ventilation was achieved with a small‐animal ventilator (RWD Life Science). The heart was exposed via left thoracotomy at the third and fourth intercostal space, and the left anterior descending coronary artery was ligated at 2–3 mm distance from the origin place between the pulmonary artery conus and the left atrium. The ligation material was a 6–0 polypropylene ligature. Electrocardiogram (ECG) data showed ST segment elevation, decreased of anterior wall motion of the left ventricle (LAD), and a mottled and pale appearance at the infarct areas, confirming the success of the infarction (Figure S1A, Figure 1B). In the sham group, the rat heart was exposed, but only threading, without ligation of the left coronary artery.

### 2.2 PVN microinjection

PVN microinjection followed the previously reported procedures.^21^ In brief, rats were secured in a stereotaxic apparatus (RWD Life Science) and cannulas were implanted in the bilateral PVN. The PVN coordinates were determined at 1.8 mm posterior, 0.4 mm lateral to the midline and 7.9 mm beneath the skull surface. A total volume of 1.0 μl microinjector was connected to the cannula with a PE‐10 tube, and 50 nl control shRNA or METTL3 shRNA was injected into the PVN using a microinjector as designed. The injection rate was set at 0.1 μl/min by using an infusion pump. The microinjector was kept on site for 15 min to allow shRNA diffusion and avoid shRNA spillover. The microinjector was then removed. PVN location in rats was determined by microinjection of methylene blue^35^ (Figure S2A).

### 2.3 Experimental design

Three independent experiments were conducted as following described.

### 2.4 Protocol 1

Forty‐five of fifty surviving rats were divided into five groups (sham, MI after 1, 3, 5 and 7 days; *n* = 9 per group). The temporal expression of m^6^A quantification in the total m^6^A level of PVN was detected using antibody‐based colorimetric methods. The PVN total RNA and proteins were extracted for m^6^A‐related enzymes detection using quantitative real‐time PCR (RT‐PCR) and Western blotting.

### 2.5 Protocol 2

Twenty‐four rats were randomly classified into two groups (sham, MI after 3 days; *n* = 12 per group). Subsequently, the PVNs were collected for RNA sequencing and m^6^A MeRIP sequencing.

### 2.6 Protocol 3

One hundred ninety rats were randomly divided into four groups: sham + AAV2 with a green fluorescent protein (GFP) reporter and scrambled RNA, which served as the blank control for the shRNA virus (shCtrl, *n* = 38); sham + AAV2 with shRNA for METTL3 (shMETTL3, *n* = 41); MI + shCtrl (*n* = 55); and MI + shMETTL3 (*n* = 56). To improve the gene silencing efficiency, the following three RNAi sequences targeting METTL3 were used in this study: (a) 5′ GCTACCGTATGGGACGTTAAC 3′; (b) 5′ GGACTTAAGGAATCCAGAAGC 3′; and (c) 5′ GCACATCCCACTCTGTAACC 3′. The adeno‐associated virus AAV2 was constructed by Genomeditech company (Shanghai, China), and AAV2 GFP titre was 1.0 × 10^13^ genomic particles/ml. Inhibition of METTL3 expression in the PVN was confirmed by immunofluorescence localization of GFP expression 3 weeks after injection (Figure S2B). MI surgery was performed 3 weeks after AAV microinjection in the PVN.

### 2.7 Heart rate variability measurements and cardiac echocardiography

A telemetry electrocardiograph transmitter (TR50B; ADInstrument) was positioned in the abdominal cavity of rats of as previously described.^21^ Two leads were placed on the dorsal surface of the xiphoid process and into the anterior mediastinum, close to the right atrium. Twenty‐four‐hour dynamic electrocardiography was performed, and the electrocardiogram (ECG) data were continually recorded by a PowerLab physiology system and analysed using LabChart Pro software (ADInstruments). A 15 min ECG recording was selected to analyse HRV. Low‐frequency (LF; 0.05–0.75 Hz) and high‐frequency (HF; 0.75–2.5 Hz) bands were recorded, and the ratio of LF to HF bands was calculated. Telemetric recordings were conducted for 3 days before the devices were removed. After the rats were anaesthetized, cardiac function was measured by echocardiography machine (Fujifilm Vevo 3100). The left ventricular ejection fraction (EF%) and fractional shortening (FS%) were calculated using the M‐mode recording of the parasternal long‐axis view.

### 2.8 RSNA recording

Renal sympathetic nerve activity (RSNA) recording was performed as previously described.^34^ In brief, the left renal sympathetic nerves were separated using fine glass needles, and the distal terminus of the renal nerve was displaced. The central part of the nerve was placed on a pair of platinum electrodes, and the RSNA was then recorded using power‐lab (ADInstruments). Background noise was detected after the renal sympathetic proximal section, and the experimental data baseline was corrected. The baseline level of RSNA was defined as 100% from the absolute value after subtracting the noise level. The data were analysed using LabChartPro software (AD Instruments).

### 2.9 Programmed electrical stimulation

The rats underwent ventricular programmed electrical stimulation (PES) to acquire VA susceptibility. The PES protocol was as described in our previous study.^36^ The rats were anaesthetized, and electrocardiography was performed with three electrodes placed on the upper limb and the right legs. A specially modified electrode was used to stimulate the left ventricle to study the incidence of VAs. Standard PES protocols were performed as follows: burst (cycle length 100 ms, S0), single (S1), double (S2) and triple (S3) additional stimuli. The total PES protocol lasted for 10 minutes. The arrhythmia scoring was measured according to a previous report.^37^

### 2.10 Tissue collection

The rats were sacrificed via an overdose injection of 3% sodium pentobarbital. The heart and the whole brains were removed. Extending the myocardium from the infarction scar to 0.5–1.0 mm was considered to represent the infarcted myocardium. The heart was excised, frozen in liquid nitrogen, then laterally sliced into 2 mm thick slices and incubated in 2% 2,3,5‐triphenyltetrazolium chloride (TTC; Solarbio, Beijing, China) at 37°C for 30 min. After TTC staining, the surviving myocardium was red, and the infarct area was white. Image J software was performed for calculating infarct size as a percentage of LV. Rats with infarct size greater than 30% were selected based on clinical importance. Two different methods were used to prepare brain tissue for further study: (1) The PVN was collected from the entire brain tissue and stored at −80°C for biochemical analysis. (2) Entire brain tissues were fixed in 4% paraformaldehyde at 4°C for 24 h, then embedded in paraffin, and cut into 5 μm thick sections for immunohistochemistry and immunofluorescence. Blood samples were collected, and the serum was collected by centrifugation (1,200 × *g*, 20 min) at 4°C, and immediately stored in a −80°C freezer for future ELISA tests.

### 2.11 m^6^A quantification

RNA‐easy™ Isolation Reagent (Vazyme Biotech Co.) was used to extract total RNA from the PVN. m^6^A methylated RNA was measured by using an m^6^A RNA Methylation Quantification Kit (Colorimetric; EpiGentek). In brief, a sample of total RNA (200 ng) was used to the specifically capture and detect antibody binding. The m^6^A signal was quantified at a wavelength of 450 nm.

### 2.12 Quantitative real‐time PCR analysis

Total PVN RNA was extracted using the RNA‐easy™ Isolation Reagent (Vazyme Biotech Co.). A PrimeScript™ RT kit (Vazyme Biotech Co.) was used for reverse transcription. Relative mRNA level was detected by in a Bio‐Rad iQ5 Multicolor Real‐Time PCR system (Bio‐Rad Laboratories, CA, USA) with SYBR Green (Vazyme Biotech Co.). The 2^−ΔΔ^ *^C^* ^t^ method was used to calculate the relative targeted gene expression and normalized to GAPDH expression. Primer sequences were listed in Table 1.

**TABLE 1:** Primers used for quantitative real‐time PCR and MeRIP‐qPCR

Gene	Primer	Gene sequence
METTL3	Forward Reverse	CCTCAGATGTTGACCTGGAGATAG GACTGTTCCTTGGCTGTTGTG
METTL14	Forward Reverse	GATCGCAGCACCTCGGTCATT CCCACTTTCGCAAACATACTCTCC
WTAP	Forward Reverse	TTCAAACGATGTGACTGGCTTA TCTCCTGTTCCTTGGTTGCTA
FTO	Forward Reverse	TGTGGAAGAAGATGGAGAGTGTGA GGATCAGGACGGCAGACAGAA
ALKBH5	Forward Reverse	GGGTTCTTATGTTCTTGGCTTTCC ATCTCTACTGGCTACTCTGGTGT
TLR4	Forward Reverse	AGAATCTGGTGGCTGTGGAG CTTGGGCTTGAATGGAGTCA
GAPDH	Forward Reverse	GGCACAGTCAAGGCTGAGAATG ATGGTGGTGAAGACGCCAGTA

### 2.13 RNA‐seq and m^6^A MeRIP‐seq

RNA sequencing and MeRIP sequencing were performed simultaneously (Sinomics Corporation). The PVN tissues were collected, and total RNA was extracted by TRIzol reagent (Invitrogen).

RNA‐seq: Strand‐specific libraries were prepared using a TruSeq^®^ Stranded Total RNA Sample Preparation kit (Illumina, CA, USA) according to the manufacturer's instructions. An Illumina HiSeq X Ten machine (Illumina) was used to perform RNA‐seq. Data analyses were performed as previously described.^38^

MeRIP‐seq: The RNA was segmented and incubated with m^6^A antibody (1:1000,202003, Synaptic Systems, Germany). Sequencing was conducted on an Illumina Novaseq™6000 machine (LC‐Biotechnology Co.). Data analyses were performed as previously described.^39^

### 2.14 Methylated immunoprecipitation‐qPCR (MeRIP‐qPCR)

Experiments were performed as previously described.^40^ Total RNA was extracted from PVNs and then conjugated in IP buffer with protein A/G magnetic beads, containing m^6^A antibody (1:1,000, Synaptic Systems) and anti‐IgG, supplemented with RNase inhibitor and protease inhibitor overnight at 4°C. Relative enrichment was calculated using the 2^−ΔΔ^ *^C^* ^t^ method compared with the input sample. Primer sequences for MeRIP‐qPCR were presented in Table 1.

### 2.15 Western blotting

Nuclear and cytoplasmic protein isolation from the PVN was performed using a nuclear and cytoplasmic extraction kit (Beyotime Biotechnology). Nuclear proteins were used for NF‐κB p65 expression analysis, and cytoplasmic proteins were used for METTL3, METTL14, WTAP, FTO, ALKBH5, TLR4 and IκB‐α analysis. The protein lysate concentration was measured using a BCA kit (Elabscience). Proteins (approximately 50 µg) were subjected to 6%–15% SDS‐polyacrylamide gels and transferred onto polyvinylidene difluoride membranes (PVDF). After blocking with 5% non‐fat milk diluted in TBST for 1 h, and the membranes were then incubated with different primary antibodies at 4°C overnight: anti‐METTL3 rabbit monoclonal (1:1000, Abcam), anti‐METTL14 rabbit monoclonal (1:1000, Cell Signaling Technology), anti‐WTAP mouse monoclonal (1:1000, Proteintech), anti‐FTO rabbit polyclonal (1:500, GeneTex), anti‐ALKBH5 rabbit monoclonal (1:1000; Abcam), anti‐TLR4 rabbit polyclonal (1:500; GeneTex), anti‐IκB‐α rabbit polyclonal (1:1000; Abcam), anti‐NF‐κB p65 rabbit monoclonal (1:1000; Cell Signaling Technology), anti‐Histone 3 rabbit monoclonal (1:1000, Bo Ao Sen Biotechnology Co.) and anti‐GAPDH rabbit monoclonal (1:1000, Cell Signaling Technology) antibodies. The membrane was then incubated with HRP‐conjugated goat anti‐rabbit or goat anti‐mouse secondary antibodies (1:5000, Absin) at room temperature for 2 h. Protein bands were detected using an ECL chromogenic substrate (Millipore) and analysed using a Chemiluminescence apparatus (Bio‐Rad).

### 2.16 Immunohistochemistry

The expression of central sympathetic proteins was detected by immunohistochemistry. Central sympathetic activity was measured by Fos family assays after MI. Fos family proteins were quantified using a mouse monoclonal anti‐c‐Fos (E‐8) antibody, which recognizes c‐Fos, Fos‐B, Fra‐1 and Fra‐2 proteins. Tissue slices were probed with the primary antibody c‐Fos (E‐8) mouse monoclonal antibody (1:50, Santa Cruz, CA, USA) overnight at 4℃. Sections were incubated with DAB substrate (ZSGB‐BIO; Beijing, China) and then counterstained with haematoxylin. Images were obtained using an LCX100 imaging system (Olympus). The number of METTL3 and Fos‐positive cells in the bilateral boundary of the PVN was analysed by Image‐Pro Plus 6.0.

### 2.17 Immunofluorescence staining

Immunofluorescence staining was performed by using a multiplex immunofluorescence staining kit (abs50012, Absin) as previously described.^41^ The tissue sections were dewaxed in xylene, dehydrated in alcohol, repaired by microwave and blocked with 5% goat serum. The sections were incubated with primary antibody anti‐iba1 rabbit monoclonal (1:2000, Abcam) at room temperature for 1 h. The secondary antibody was incubated for 10 min and incubated with monochromatic fluorescent dyes 520 for 10 min, and then, microwave repair was cooled to room temperature. The above steps were repeated to complete the staining of anti‐METTL3 rabbit monoclonal (1:500, Abcam). We used 570 as a fluorescent dye for METTL3. Finally, the sections were stained with DAPI and anti‐fluorescence quencher seal. Images of stained sections were obtained by confocal microscope (Nikon A1+). The number of METTL3 or iba1 positive cells was calculated using Image‐Pro Plus 6.0.

### 2.18 Enzyme‐linked immunosorbent assay (ELISA)

The levels of IL‐1β, TNF‐α and the excitatory neurotransmitter norepinephrine (NE) were detected using an ELISA kit (Elabscience). The OD value at 450 nm was used to determine protein concentrations by the end. NE in serum was recorded in pg/ml, and myocardium tissue concentrations of NE were recorded in pg/mg.

### 2.19 Statistics analysis

Data are presented as the mean ±SD. GraphPad Prism 8.0 (GraphPad Software) was used to analyse the data. Two‐tailed unpaired Student's t tests were used for two groups. Two‐way analysis of variance (ANOVA) followed was performed to analyse the three or more groups. *p* < 0.05 was considered statistically different.

## 3 RESULTS

### 3.1 m^6^A and METTL3 were upregulated in the PVN post‐MI

To uncover the expression properties of m^6^A modification and m^6^A regulators in the PVN after MI, m^6^A level in total RNA, mRNA and protein levels of METTL3, METTL14, WTAP, FTO and ALKBH5 were measured as shown. Figure 1A showed the diagram of the experiment. Compared with the sham group, the total m^6^A levels in the PVN began to upregulate at 1day post‐MI (*p* > 0.05, not significant) and significantly increased from 3 days to 7 days post‐MI (Figure 1B). As one of the methyltransferases that regulate m^6^A installation, METTL3 slightly increased at 1day post‐MI (*p* > 0.05, not significant) and significantly elevated in mRNA levels from 3 days to 7 days post‐MI compared with the sham group (Figure 1C). FTO, the demethylase that was responsible for m^6^A removal, showed a significant decrease in mRNA levels from 3 to 7 days post‐MI compared with the sham group (Figure 1E). The protein expression levels of METTL3 and FTO were consistent with their mRNA levels, respectively (Figure 1D, F). The other methyltransferases, METTL14 and WTAP, and demethylase, ALKBH5 showed no differences among all the groups at either the mRNA or protein levels (Figure S3). To address the correlation between total m^6^A levels and these m^6^A regulators, Pearson correlation analysis was performed with qRT‐PCR data, and a correlation heatmap was generated (Figure 1G). The expression of total m^6^A and METTL3 was positively correlated, and FTO expression was negatively correlated as shown in Figure 1G. To date, the upregulated METTL3 as well as the downregulated FTO contributed to increased installation of m^6^A on RNA of PVN after MI. In our study, the expression levels of m^6^A and METTL3 were significantly increased 3 days after MI. Previous studies have shown that the abnormal sympathetic nerve function is mainly manifested in its activity at the early phase of AMI (1–4 days).^21^ When AMI occurs, an acute inflammatory response occurs approximately 4h later, significantly enhanced expressions of TNF‐α and IL‐1β in the myocardium. High inflammatory response can still be detected 2–3 days after AMI, which gradually decreases to normal low level after about 2 weeks.^42^ In summary, we chose 3 days after MI for further study.

### 3.2 TLR4/NF‐κB signalling pathway as the downstream target of m^6^A modulation in the PVN

**FIGURE 2:**
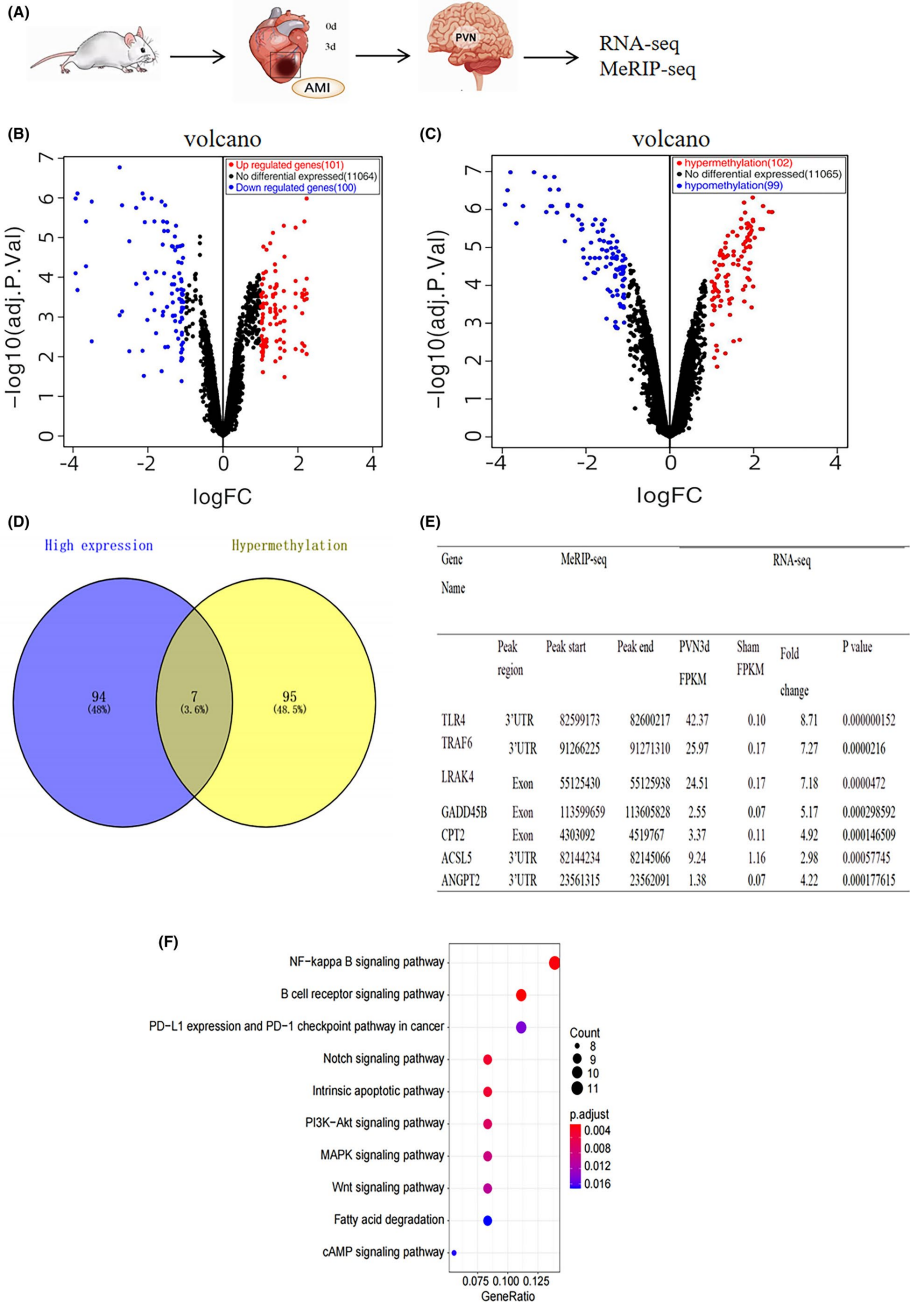
Conjoint analysis of RNA‐seq and MeRIP‐seq data in the PVN 3 days post‐MI. (A) Diagram of the experiment. (B) The differentially expressed genes between sham and MI groups (3 days) were shown in Volcano plots with statistical significance by RNA‐seq. Red dots represent significantly upregulated genes (log2 Fold Change >1 and *p *< 0.05); blue dots represent significantly downregulated genes (log2 Fold Change ≤1 and *p *< 0.05); black dots indicate the genes with no changes. (C) The methylation of genes that differentially expressed between the sham and MI group (3 days) were shown in Volcano plots with statistical significance by MeRIP‐seq. Red dots represent significantly hypermethylated genes (log2 Fold Change >1 and *p *< 0.05); blue dots represent significantly hypomethylated genes (log2 Fold Change≤1 and *p *< 0.05); black dots indicate the genes with no changes. (D) Venn diagrams showing the overlap between hypermethylated genes with m^6^A modifications and genes that were shown to be highly expressed. (E) Seven genes in the intersection of hypermethylated genes and highly expressed genes are listed in the table. Fold change in TLR4 ranked first among the seven genes and m^6^A modification occurred at the 3'UTR. (F) Bubble charts showing the top 10 significantly enriched pathways of hypermethylated genes in the sham and MI groups (3 days) by KEGG analysis of the biological pathways. The colour scale at the top right represents the number of genes, and the size of the circle on the lower right represents the relative expression level of the genes. The results from the ClusterProfiler analyses were considered enriched at a *p* < 0.05 and an FDR of 0.05. *n* = 12 per group. Abbreviation: MI, myocardial infarction

To define the potential downstream targets and associated pathways for METTL3‐mediated m^6^A modification in the PVN at day 3 of MI, RNA‐seq and m^6^A MeRIP‐seq were performed. Figure 2A showed a diagram of the experiment. A total of 11265 genes were found and plotted in the volcano graph (Figure 2B) using RNA‐seq, in which 101 and 100 genes were found to be significantly upregulated and downregulated, respectively, comparing with the sham group (log2 Fold Change >1 and *p *< 0.05; log2 Fold Change ≤1 and *p *< 0.05). Regarding to the methylation level of genes, 100 genes were significantly hypermethylated (log2 Fold Change >1 and *p *< 0.05) and 99 genes were significantly hypomethylated (log2 Fold Change ≤1 and *p *< 0.05) by m^6^A MeRIP‐seq analysis (Figure 2C). To find out methylation state of these highly expressed genes, we plotted a Venn diagram to demonstrate the cross‐section of highly expressed genes and hypermethylated genes with m^6^A (Figure 2D). Seven genes were highly expressed and hypermethylated, accounting for 3.6% of the total analysed genes. The seven genes were listed in Figure 2E. Fold change of TLR4 ranked the first in the seven genes and m^6^A modification occurred at 3'UTR (Figure 2E) indicating the pathophysiological significance of TLR4 gene. To define the biological function of m^6^A modification, KEGG pathway was analysed (Figure 2F). The enriched hypermethylated genes were predominantly related to the NF‐κB signalling pathway.

### 3.3 METTL3‐mediated m^6^A modification activated TLR4/NF‐κB signalling pathway in the PVN

**FIGURE 3:**
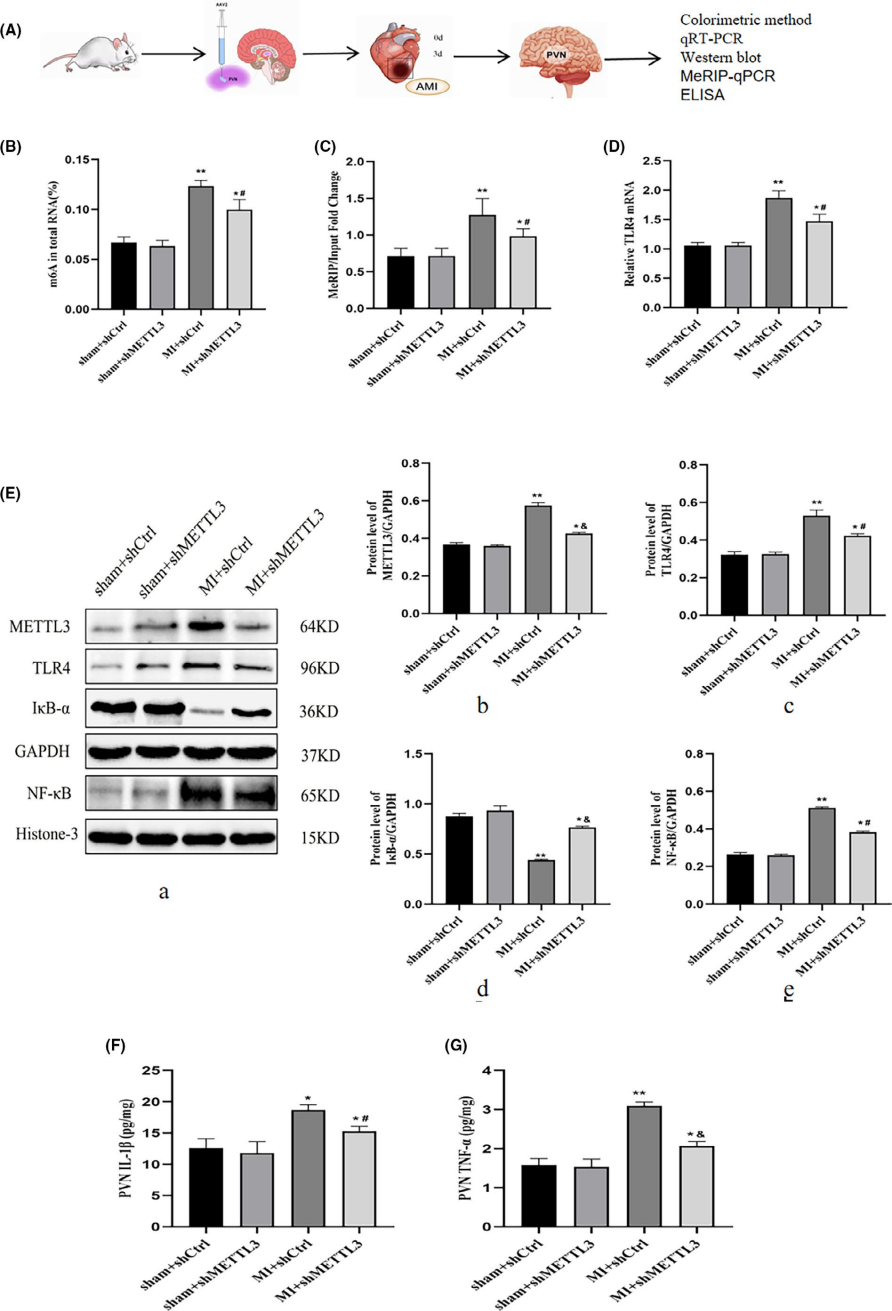
METTL3‐mediated m^6^A promoted TLR4/NF‐κB signalling in the PVN post‐MI. (A) Diagram of the experiment. (B) The total m^6^A levels were detected by colorimetric methods. (C) MeRIP‐qPCR showed m^6^A modification occurred in the TLR4 3'‐UTR region. (D) TLR4 mRNA expression was detected by qRT‐PCR. (E) Representative protein expression levels of METTL3, TLR4, IκB‐α and NF‐κB as determined by Western blotting. GAPDH served as the internal control for normalizing the relative expression of METTL3, TLR4 and IκB‐α, while histone 3 served as the internal control for NF‐κB. The cytokine levels of IL‐1β (F) and TNF‐α (G) from the PVN tissue as measured by ELISA. n=28–30 per group. Data are presented as mean ± *SD*. *p < 0.05 and **p < 0.01 versus sham groups; ^#^ *p* < 0.05 and ^&^ *p* < 0.01 versus MI + shCtrl group. Abbreviations: MI, myocardial infarction; PVN, paraventricular nucleus; SD, standard deviation

To explore the pathophysiological significance of METTL3‐mediated m^6^A modification in the TLR4/NF‐κB pathway, AAV‐shRNA was microinjected into the PVN. Figure 3A showed the diagram of the experiment. m^6^A levels in the PVN were increased in the MI + shCtrl group; however, they were reduced after METTL3 knockdown in the MI + shMETTL3 group (Figure 3B). m^6^A modification occurred in the TLR4 3'‐UTR region, as confirmed by MeRIP‐qPCR. The data showed that downregulating METTL3 decreased m^6^A levels of TLR4 mRNA after MI (Figure 3C). TLR4 mRNA expression was decreased in the MI + shMETTL3 group by qRT‐PCR. (Figure 3D). Western blotting revealed that infarction induced higher protein expression of METTL3 and TLR4 in the cytoplasm, and increased NF‐κB and decreased IκB‐α levels in nuclei (Figure 3E). IL‐1β and TNF‐α were found to be participated in NF‐κB activation in rat PVN post‐MI. Higher levels of IL‐1β and TNF‐α were observed by ELISA in the MI + shCtrl group, while IL‐1β and TNF‐α levels were significantly decreased in rats treated with MI + shMETTL3 (Figure 3F, G) which further confirmed that MI‐induced NF‐κB nuclear translocation via an IκB‐α‐dependent manner. Moreover, METTL3 knockdown significantly suppressed IκB‐α degradation and the nuclear translocation of NF‐κB, together with the decreased IL‐1β and TNF‐α levels. These findings indicated that METTL3 activated TLR4‐NF‐κB signalling in an m^6^A‐dependent manner in the PVN after MI and promoted secretion of inflammatory cytokines.

### 3.4 METTL3 was mainly expressed in microglia of PVN post‐MI

**FIGURE 4:**
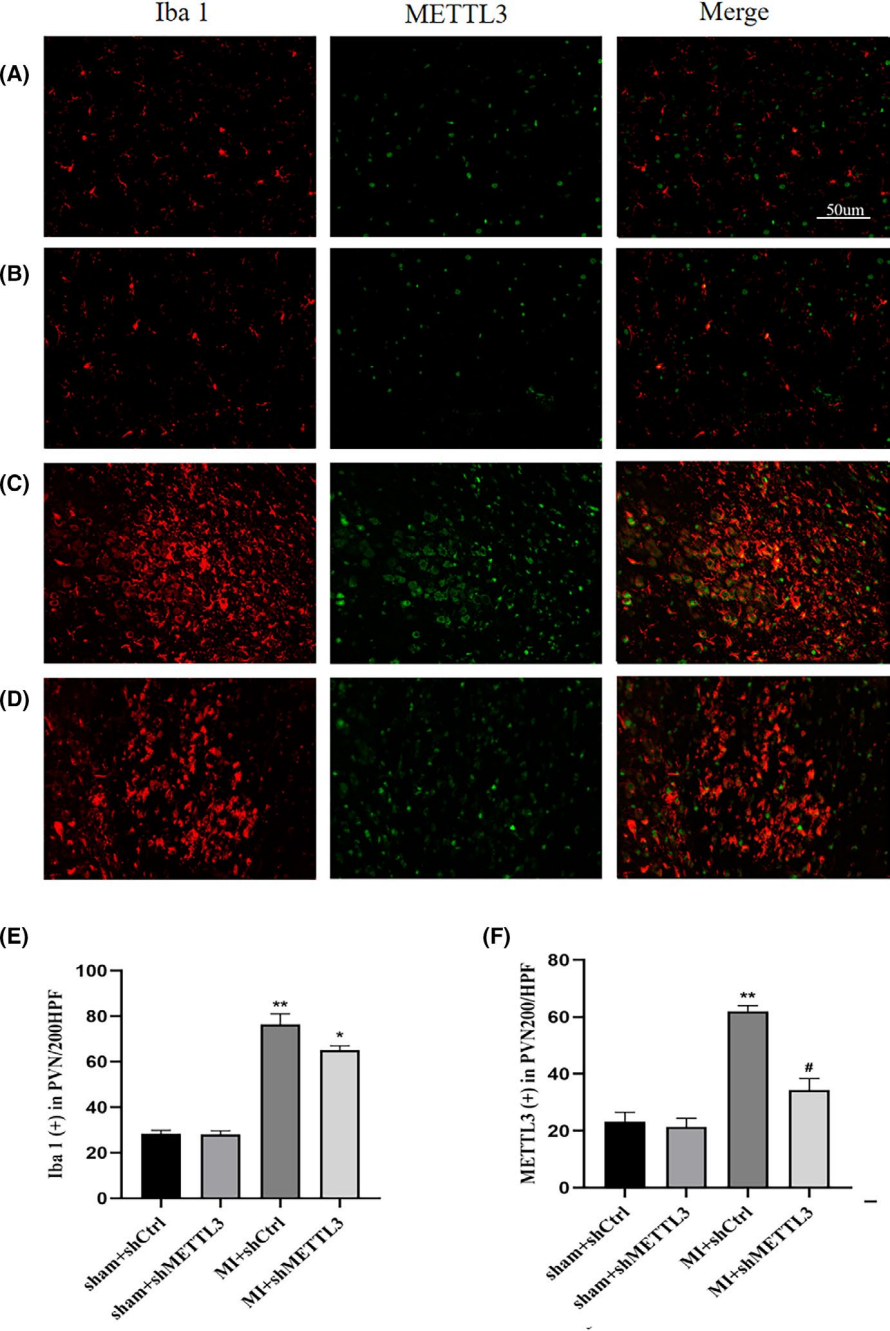
METTL3 was highly expressed in microglia of PVN post‐MI. Representative double‐immunostaining of Iba‐1 (red), METTL3 (green) in the PVN from sham + shCtrl (A), sham+shMETTL3 (B), MI+shCtrl (C) and MI+shMETTL3 (D) groups (magnification 200×). Bar =50μm. Quantitation of Iba 1‐positive(E)and METTL3 positive(F)cells. *n* = 10–12 per group. Data represent the mean ±SD. **p < 0.01 and *p < 0.05 versus sham groups; ^#^ *p* < 0.05 versus MI + shCtrl group. Abbreviations: MI, myocardial infarction; PVN, paraventricular nucleus; SD, standard deviation

Double staining of Iba 1 (red) and METTL3 (green) using immunofluorescence was performed to locate METTL3 in microglia (Figure 4). Iba 1‐positive staining was measured to quantify the degree of inflammatory microglial infiltration after MI. Higher Iba 1 expression was observed in the MI groups (Figure 4C, D, E). However, Iba 1 was slightly attenuated in the MI + shMETTL3 group compared with the MI + shCtrl group (*p* > 0.5, Figure 4D, E, not significant). But METTL3 was increased significantly in the MI + shCtrl group and decreased in the MI + shMETTL3 group (Figure 4D, F). In the initial stages of MI inflammation in rats, almost all METTL3 was expressed in microglia in the PVN region.

### 3.5 METTL3 inhibition attenuated sympathetic hyperactivity and improved cardiac function post‐MI

**FIGURE 5:**
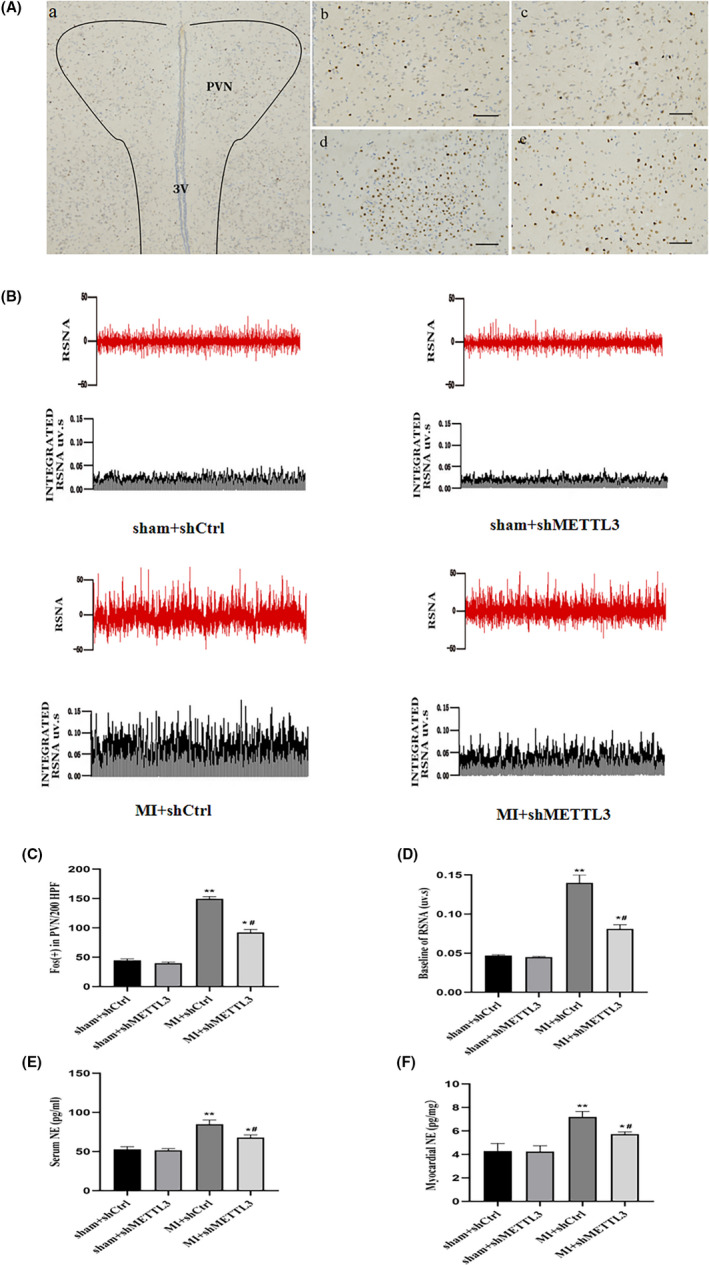
METTL3 inhibition attenuated central and peripheral sympathetic nerve activity. (A) Representative immunohistochemical images of central sympathetic nerve activity in the PVN (magnification 200×). Fos family proteins, the markers of neuronal activation, were shown as brown puncta. Nuclei (blue) were stained with haematoxylin. (a) A lower magnification of the PVN, (b) sham + shCtrl, (c)sham + shMETTL3, (d) MI + shCtrl and (e) MI + shMETTL3 groups. Bar = 50 μm. (B) Typical recordings from the left renal RSNA, integrated RSNA. (C) Quantitation of Fos‐positive cells. (D) Baseline of RSNA. Cytokine levels of NE in serum (E) and cardiac tissue (F) were measured by ELISA. n = 10–12 per group. Data are presented as mean ±SD. **p < 0.01 and *p < 0.05 versus sham groups; ^#^ *p* < 0.05 versus MI + shCtrl group. 3V, 3rd ventricle; Abbreviations: MI, myocardial infarction; NE, norepinephrine; PVN, paraventricular nucleus; RSNA, renal sympathetic nerve activity; SD, standard deviation

To investigate the functional roles of METTL3 in sympathetic nervous activity, the relevant measurements of the central and peripheral nervous systems were quantified. Figure 5A‐a depicted a representative photomicrograph of the PVN. Fos, indicator of neuronal activation. Fos was increased in the MI + shCtrl and MI + shMETTL3 groups compared with sham + shCtrl and sham + shMETTL3 groups (Figure 5A‐b‐e, C). Moreover, Fos expression was decreased following the infection of METTL3 shRNA comparing with MI+shCtrl group (Figure 5A‐d‐e, C). Fos was used to reveal the role of METTL3 in central neuronal activity, which was a prerequisite for peripheral sympathetic hyperactivity. Notably, the neural factors that activate neuronal excitation in the PVN or reduce the inhibitory neurotransmission are evidenced to be pivotal for sympathetic excitation.^43^

**FIGURE 6:**
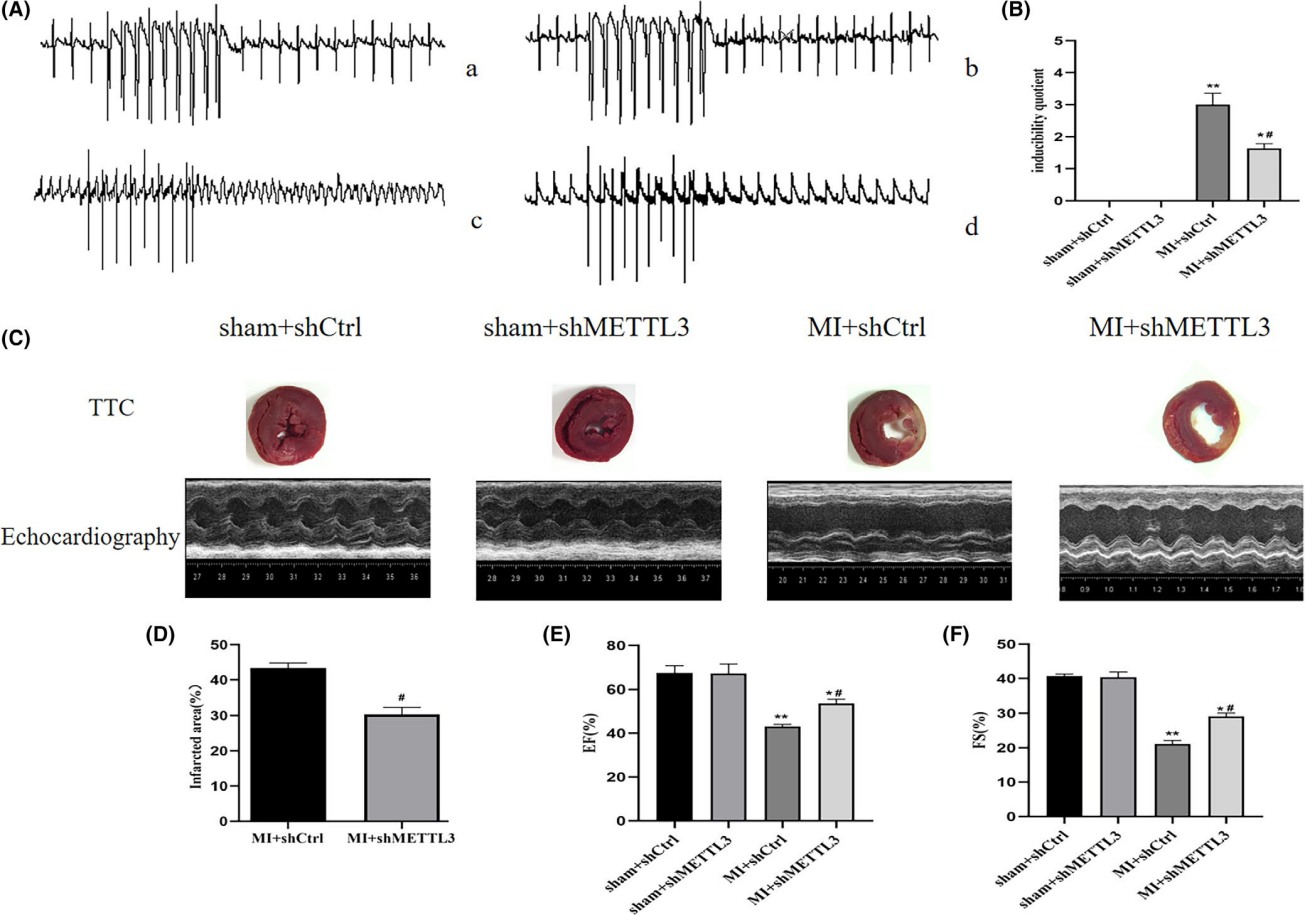
Programmable electrical stimulation at and cardiac function were measured at 3 days post‐MI. (A) Representative electrocardiogram of programmable electrical stimulation at 3 days post‐MI in the (a) sham+shCtrl, (b) sham+shMETTL3, (c) MI+shCtrl and (d) MI+shMETTL3 groups. (B) Comparison of arrhythmia scores between the four groups. (C) TTC staining and parasternal long axis view of M‐type echo of rat heart in sham+shCtrl, sham+shMETTL3, MI+shCtrl and MI+shMETTL3 groups. (D) Comparisons of the infarcted area in the MI+shCtrl and MI+shMETTL3 groups. Comparisons of the left ventricular EF (E) and left ventricular FS (F) between the four groups. n=18–20 per group. Data are presented as mean ± *SD*. **p < 0.01 and *p < 0.05 versus sham groups; ^#^ *p* < 0.05 versus MI + shCtrl group. Abbreviations: EF, ejection fraction; FS, fractional shortening; MI, myocardial infarction; SD, standard deviation; VA, ventricular arrhythmia

Meanwhile, to assess the peripheral sympathetic activity that was elicited by central sympathetic outflow, serum and cardiac norepinephrine (NE) were tested and left renal sympathetic nerve activity (RSNA) was recorded. Serum NE and myocardial NE were increased in the MI + shCtrl and MI + shMETTL3 groups compared with that of the sham + shCtrl and sham + shMETTL3 groups, respectively (Figure 5E, F). Serum and myocardial NE decreased in MI + shMETTL3 group compared with the MI + shCtrl group (Figure 5E, F). RSNA was increased in the MI + shCtrl and MI + shMETTL3 groups compared with the sham + shCtrl and sham + shMETTL3 groups (Figure 5B, D). RSNA was decreased in the MI + shMETTL3 group compared with the MI + shCtrl group (Figure 5B, D), indicating the significance of METTL3 in peripheral sympathetic activation. Programmed electrical stimulation (PES) indicated that the rats were vulnerable to experience the induction of VAs following MI. However, VAs score decreased significantly in MI + shMETTL3 group comparing with the MI + shCtrl group (Figure 6A‐c‐d, B) implying METTL3 knockdown had an inhibitory effect on the incidence of VAs after MI.

Besides, heart rate variability (HRV) analysis was performed to evaluate autonomic nerve control systematically. The low‐frequency (LF) component was correlated with sympathetic tone, whereas the high‐frequency (HF) component was considered a marker of parasympathetic tone. The LF/HF ratio was used as an index of the interaction between the sympathetic and parasympathetic activities. Compared with the MI +shCtrl group, the MI + shMETTL3 group showed downregulated LF component and LF/HF ratio (Table 2), indicating that METTL3 knockdown decreased sympathetic activity.

**TABLE 2:** HRV measurements data at the end of study

Parameters	Sham		Ligation	
	Sham + shCtrl	Sham + shMETTL3	MI + shCtrl	MI + shMETTL3
No. of surviving rats	38	40	45	42
HR, bpm	390 ± 10.7	409 ± 14.0	445 ± 15.1**	423 ± 11.5*^,#^
LF, nu	19.52 ± 1.19	19.74 ± 4.11	42.64 ± 6.03**	33.92 ± 3.50*^,#^
HF, nu	83.96 ± 3.61	82.17 ± 4.18	72.25 ± 5.11*	77.89 ± 4.89*
LF/HF	0.2324 ± 0.05	0.2290 ± 0.03	0.5902 ± 0.03**	0.4354 ± 0.04*^,#^

Note

Data are presented as mean ± *SD*.

Abbreviations: HF, high frequency; HR, heart rate; LF, low frequency; LF/HF, low frequency/high frequency; MI, myocardial infarction.

*p < 0.05 and **p < 0.01 versus Sham groups.

^#^ *p* < 0.05 versus MI + shCtrl group.

Myocardial infarct size and cardiac function were also analysed to determine the consequent effects of METTL3 (Figure 6C–F). As relative to the infarct size in MI + shMETTL3 group was reduced versus MI + shCtrl group (Figure 6C, D). Echocardiography data showed reduced EF and FS in both MI + shCtrl versus sham + shCtrl and MI + shMETTL3 versus sham + shMETTL3 groups (Figure 6C, E, F). METLL3 knockdown rescued EF and FS (Figure 6C, E, F). These data suggested the protective effect by targeting METTL3 in the PVN.

## 4 DISCUSSION

In the present study, we studied regulatory effects of m^6^A methylation modification in sympathetic hyperactivity and the consequent cardiac and systematic outcomes by targeting METTL3 in the PVN. The main findings were as follows: (a) Total m^6^A and METTL3 were highly expressed in rat PVN after MI, and METTL3 was primarily distributed in microglia of PVN in MI rats; (b) TLR4 mRNA expression was upregulated by METTL3‐mediated m^6^A modification and TLR4/NF‐κB served as the downstream signalling pathway in modulating central sympathetic activation initiated by MI; (c) Targeting METTL3 protected the heart from arrhythmia attack by reducing the sympathetic hyperactivity and reduced infarct size post‐MI.

Microglia, the innate immune cells in the central nervous system, are the major effector cells in response to acute and chronic neuroinflammation by producing cytokines when activated by an insult or injury. Sustained activation of microglia was observed in the PVN following MI,^21^, ^44^but not in the ventral hypothalamus or cortex,^12^ indicating that inflammation was restricted to PVN, one of the central cardiovascular regions.^45^ Besides, numerous studies have emphasized the vital roles that PVN played in kinds of cardiovascular diseases, like heart failure, myocardial ischaemia reperfusion and hypertension, via neuroinflammation.^44^, ^46^, ^47^, ^48^ Alternatively, sympathetic activation following MI was clinically correlated with VAs^49^ and has been known for decades.^50^ Although the exact mechanism of sympathetic activation is obscure, inflammation is involved in the process by evaluating cytokines released by inflammatory cells such as macrophages in local myocardial tissues and microglia in central cardiovascular regions, while inhibition of microglia activation is beneficial in reducing MI injury.^11^ Thus, targeting microglia is an efficient strategy to improve prognosis of these diseases.^51^, ^52^ In this study, we demonstrated for the first time the key roles of METTL3/m^6^A in TLR4/NF‐κB signalling mediated inflammation in microglia of PVN after MI. We also explored the potential therapeutic roles of METTL3/m^6^A by knocking down METTL3, and both the central and peripheral sympathetic activity were reduced apparently indicating that METTL3/m^6^A are the key components in microglia‐mediated inflammation in the PVN of MI. The pathophysiological roles that METTL3/m^6^A provide new perspectives for understanding the inflammatory process.

m^6^A, the most abundant internal modification in mammalian mRNA, is installed by methyltransferase complex consisting of METLL3, METTL14, WTAP, VIRMA, RBM15 and ZC3H13 and removed by FTO and ALKBH5. Clinical and basic data have shown the pro‐inflammatory^53^ or anti‐inflammatory^32^, ^33^ effect of facilized by METTL3 in human peripheral blood mononuclear and macrophage cell lines. However, little is known about the roles of METTL3/m^6^A in microglia‐mediated inflammation in PVN, especially the pathophysiological roles after MI. This study, for the first time, showed the pro‐inflammatory effect of METTL3/m^6^A through regulating TLR4 expression on microglia of PVN post‐MI. Besides METTL3, we have tested the other components of m^6^A installation on mRNA, such as METTL14 and WTAP, and found no significant difference variation among the groups. Meanwhile, FTO, the demethylase, was decreased in microglia of the PVN post‐MI.

TLR4 is a member of the TLR family that is activated by a wide range of stimulations such as LPS in brain microglia and is involved in the initiation of innate immune responses.^54^ Previous studies have shown that TLR4 is widely expressed in macrophages, hepatocytes, renal tubular cells and other cells and regulates NF‐κB signalling.^55^, ^56^ NF‐κB is a classic inflammatory factor that plays important roles in kinds of cardiovascular diseases.^57^, ^58^ NF‐κB signalling is initiated by TLR4 via the TIRAP‐MyD88 and TRIF‐TRAM pathways at different stages of NF‐κB activation.^59^ IL‐12 and TNF‐α are the two cytokines that are released through NF‐κB activation and are the TIRAP‐MyD88 pathway and TRIF‐TRAM pathways dependent, respectively. We obtained decreased TNF‐α when knocking down METTL3 in MI rats in this study, indicating that METTL3/m^6^A mediates microglia activation at least through TLR4/TRIF‐TRAM/NF‐κB pathway.

### 4.1 Outlook

We are the first to demonstrate the upregulated expression of METTL3/m^6^A clearly in the PVN of MI animal models and established the regulatory configuration of METTL3/m^6^A on sympathetic activation and arrhythmias post‐MI. The findings in this study are prominent breakthroughs in understanding the regulatory mechanism of microglia in the PVN post‐MI and provide potential therapeutic target for patients post‐MI. However, as shown in the results, targeting METTL3 can only partially decrease MI‐induced inflammation and sympathetic activation. We only tested METTL3, METLL14 and WTAP in this study, while other methyltransferases should be tested in future. Besides, contribution of demethylase was not evaluated. However, how METTL3 helps m^6^A install onto TLR4 mRNA, and the specific methylation site of m^6^A modification in TLR4 3 ‘UTR region will be investigated in future, which will assist in our understanding of microglia activation in the PVN. This study focused on the acute phase of MI, while the long‐term follow‐up of METTL3/m^6^A expression should be assessed to better qualify their functions. In addition, the roles of METTL3/m^6^A in the other cardiovascular regions, such as the rostral ventrolateral medulla (RVLM) and medial prefrontal cortex (MPFC), should be investigated in future.

### 4.2 Limitations

METTL3, as a multifunctional protein, regulated biological processes, such as mRNA translation, in addition to acting as m^6^A methyltransferase.^60^ METTL3 inhibited lipopolysaccharide (LPS)‐induced inflammation through MAPK and NF‐κB pathways in human dental pulp cells (HDPCs).^33^ We will also investigate whether METTL3 acts on other molecules or inflammatory pathways to affect sympathetic activation after MI in future. In addition, GAPDH was not a reliable normalizer for qPCR validation of genomic copy number variants because it overlaps highly homologous segmental duplications. We should be more cautious in future study. All the data sets were obtained from acute MI animal model, which differs from the natural myocardial ischaemia, we should be cautious when taking the configuration into human. Targeting METTL3 of human PVN would be challenging in future. If not, delivering shRNA‐METTL3 systemically would be out of target since METTL3 is commonly expressed in mammal cells.

## 5 CONCLUSIONS

**FIGURE 7:**
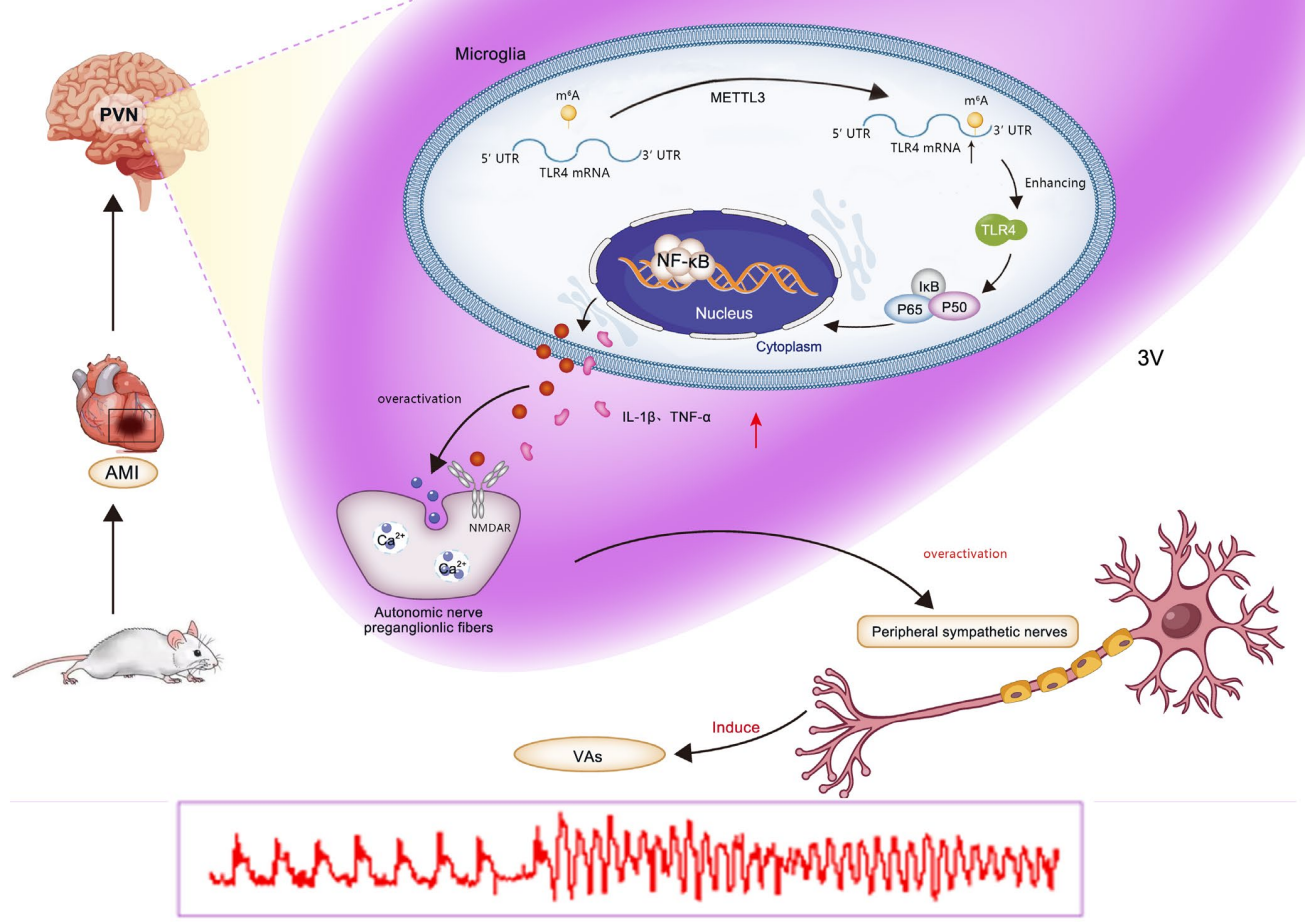
Schematic figure showing METTL3‐mediated m^6^A modification was discovered in the microglia of PVN in MI rats and enhanced m^6^A installation on TLR4 mRNA 3'UTR region was mediated by METTL3. The elevated TLR4 expression by m^6^A modification on TLR4 mRNA 3'‐UTR region combined with activated NF‐κB signalling led to the overwhelming production of pro‐inflammatory cytokines IL‐1β and TNF‐α in the PVN, thus inducing the central and peripheral sympathetic activation, and increasing the incidence of VAs after MI. Abbreviations: MI, myocardial infarction; PVN, paraventricular nucleus; VAs, ventricular arrhythmias

We demonstrated that METTL3/m^6^A was upregulated in microglia of PVN in MI rats, m^6^A installation on TLR4 mRNA 3'UTR region was increased mediated by METTL3 and enhanced TLR4 expression in the PVN combined with activated NF‐κB signalling led to the overwhelming production of pro‐inflammatory cytokines during MI, consequently, sympathetic activation initiated arrhythmia after MI (Figure 7). Targeting METTL3 attenuated VAs and protected cardiac function. METTL3 could be a potential therapeutic candidate for reducing inflammation post‐MI.

## CONFLICT OF INTEREST

The authors declare that there is no conflict of interest.

## AUTHOR CONTRIBUTIONS

**Lei Qi:** Conceptualization (equal); Investigation (equal); Writing – original draft (lead); Writing – review & editing (lead). **Hui Hu:** Methodology (supporting). **Ye Wang:** Methodology (equal); Validation (equal). **Hesheng Hu:** Validation (equal). **kang Wang:** Methodology (equal). **Pingjiang Li:** Methodology (equal). **Jie Yin:** Methodology (equal); Software (equal). **Yugen Shi:** Methodology (equal); Validation (equal). **Yu Wang:** Data curation (equal). **Yuepeng Zhao:** Methodology (equal). **Hangji Lyu:** Formal analysis (equal); Software (equal). **Meng Feng:** Formal analysis (equal). **Mei Xue:** Methodology (equal). **Xinran Li:** Methodology (equal). **Yan Li:** Conceptualization (equal); Writing – original draft (equal); Writing – review & editing (equal). **Suhua Yan:** Conceptualization (equal).

## Supporting information

Fig S1‐S3

## Data Availability

The data that support the findings of this study are available from the corresponding author upon reasonable request.
