# Supplementary material for: New insights into the central sympathetic hyperactivity post‐myocardial infarction: Roles of METTL3‐mediated m6A methylation
Source: J Cell Mol Med. 2022 Jan 17;26(4):1264–80. doi: 10.1111/jcmm.17183 (PMC8831944; doi:10.1111/jcmm.17183)
Supplement: Supplementary file 1 — Fig S1‐S3 [file JCMM-26-1264-s001.docx]

**Supplementary figures**

A


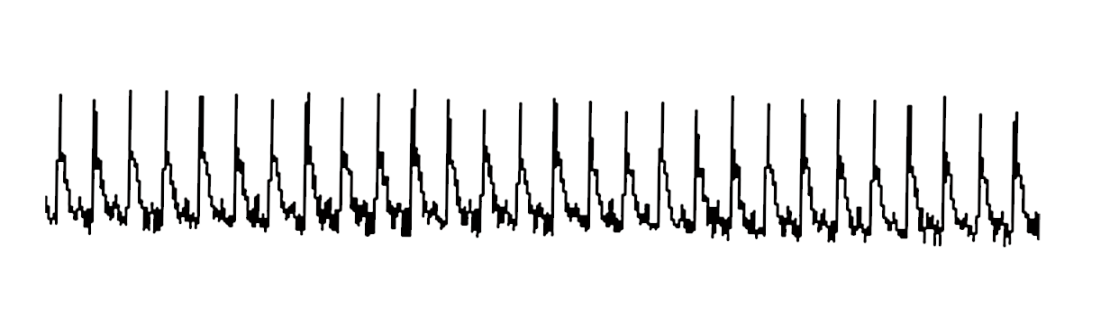


B


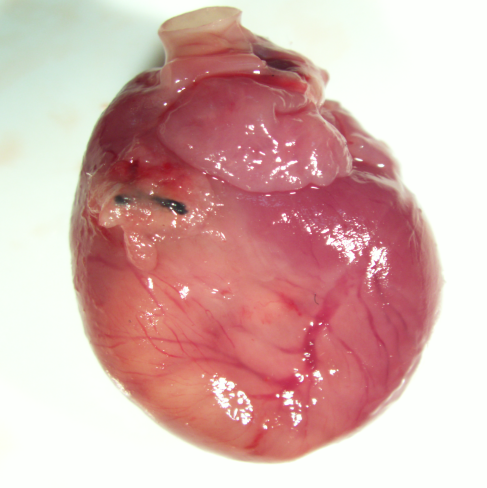


Supplementary Fig.1

A.Confirmation of myocardial infarction by recorded ECG after MI surgery: elevated ST.

B. Mottled and pale appearance of infarcted region by visualizing.

A B


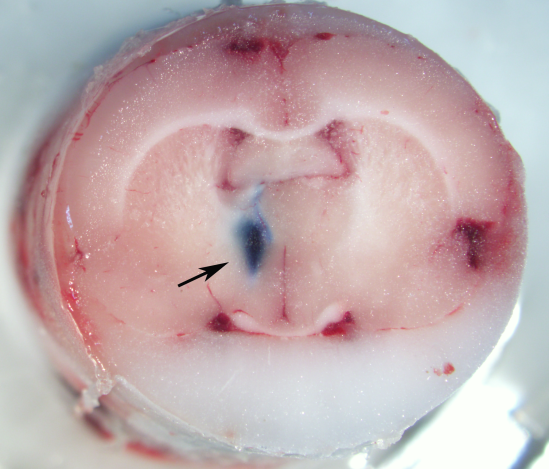

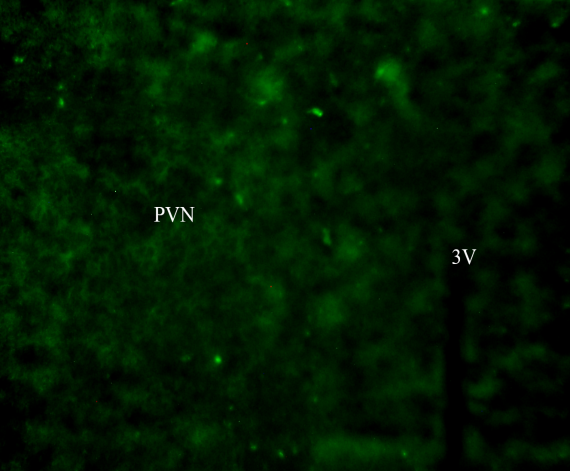


Supplementary Fig.2

A.Original color image of PVN area was delineated after injection of methylene blue. The arrow pointed to PVN area, as indicated by the blue staining.

B.Fluorescent microscopic image showing GFP expression, indicating that the shRNA complex could be efficiently delivered in vivo via PVN microinjection after 3 weeks.


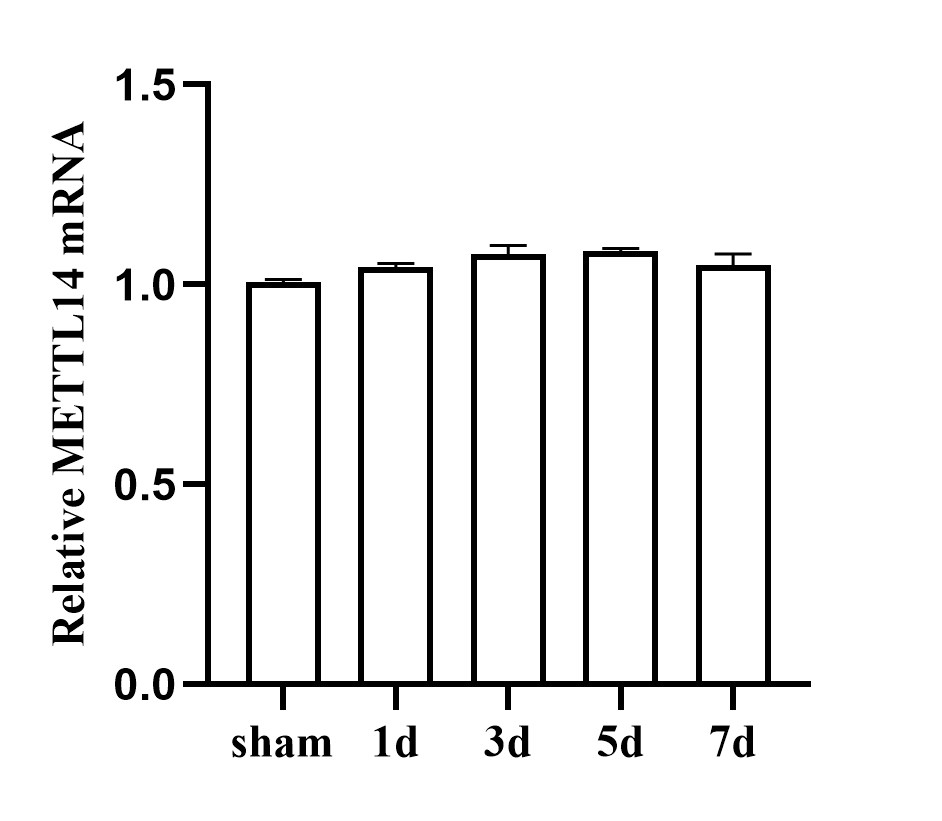

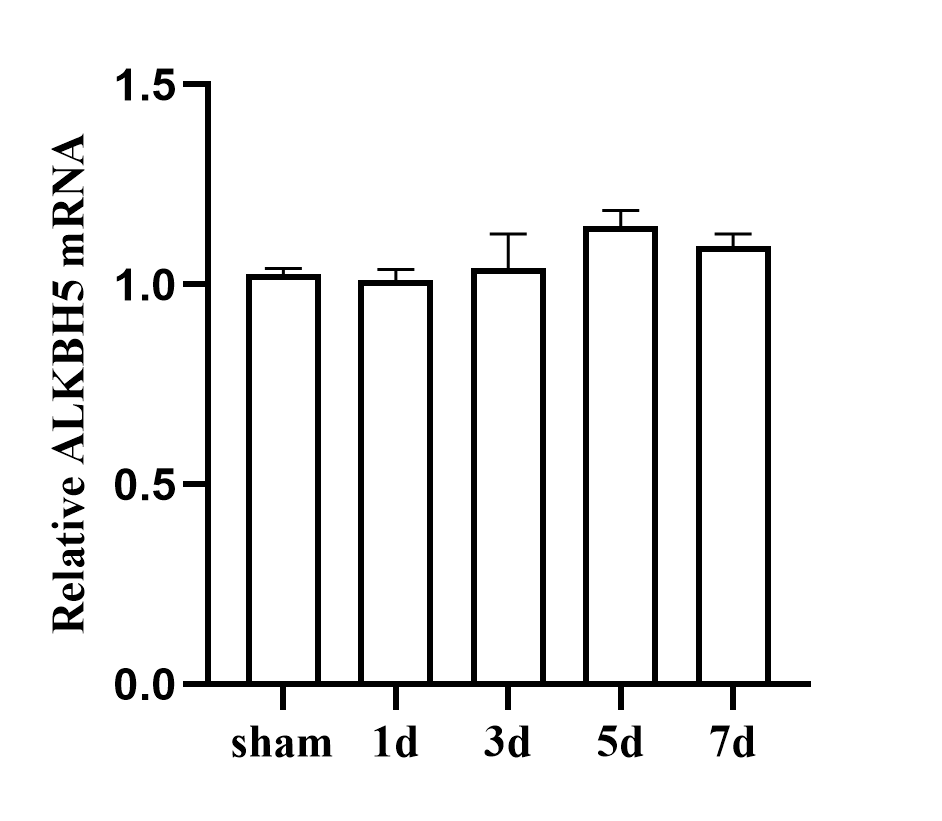

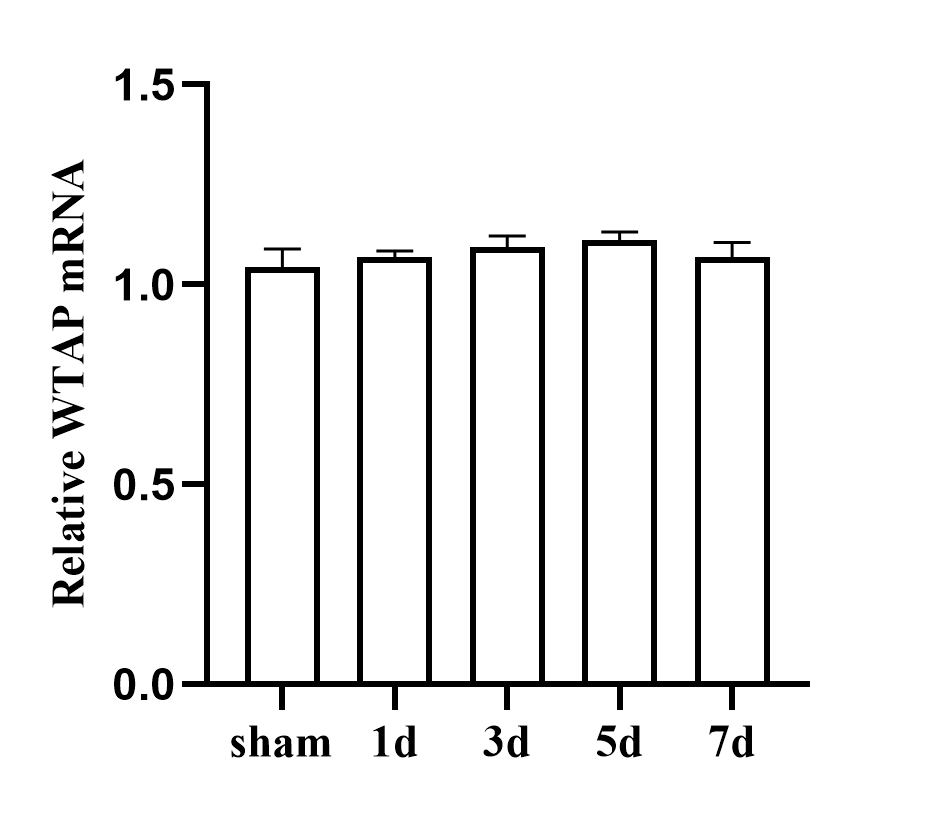
A C E


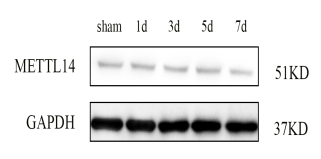

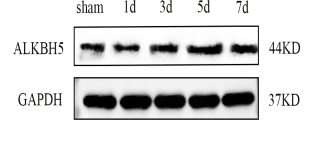

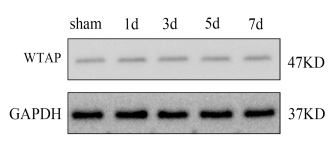
B D F


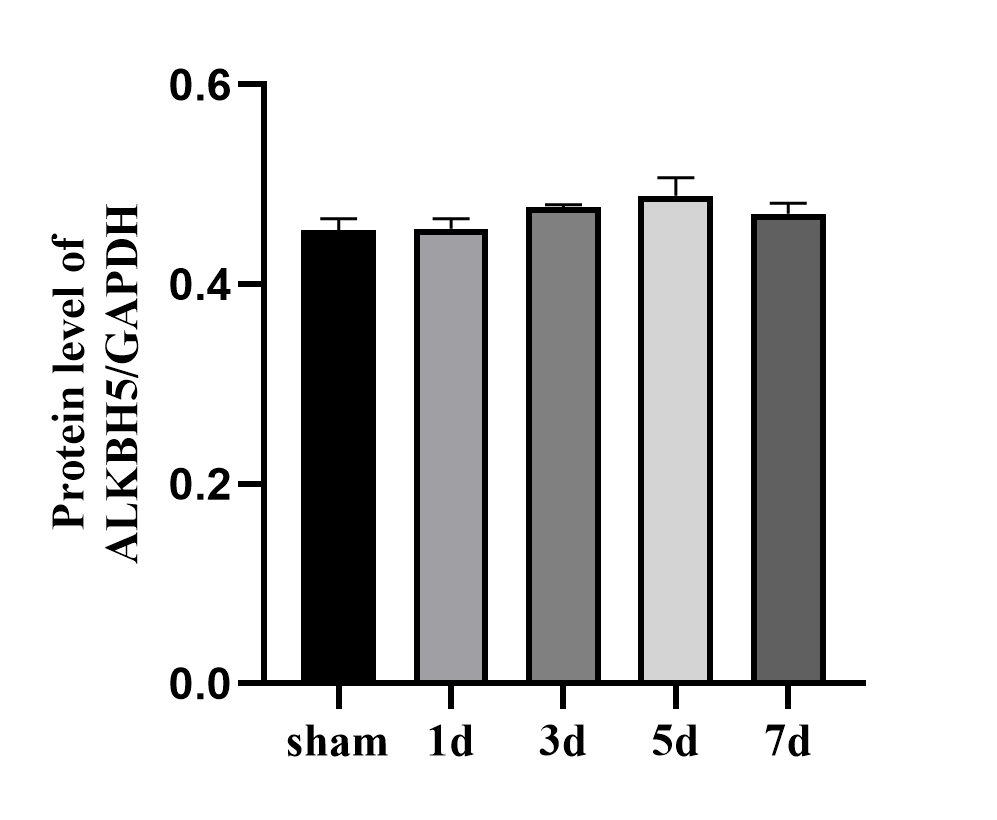

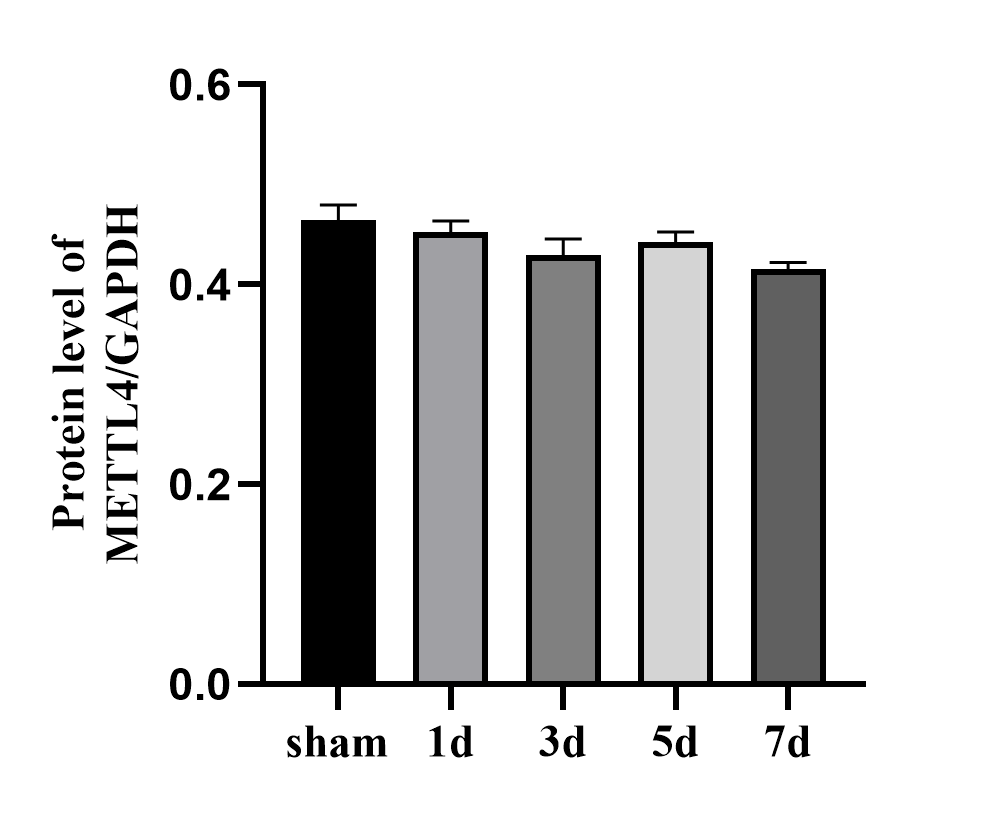


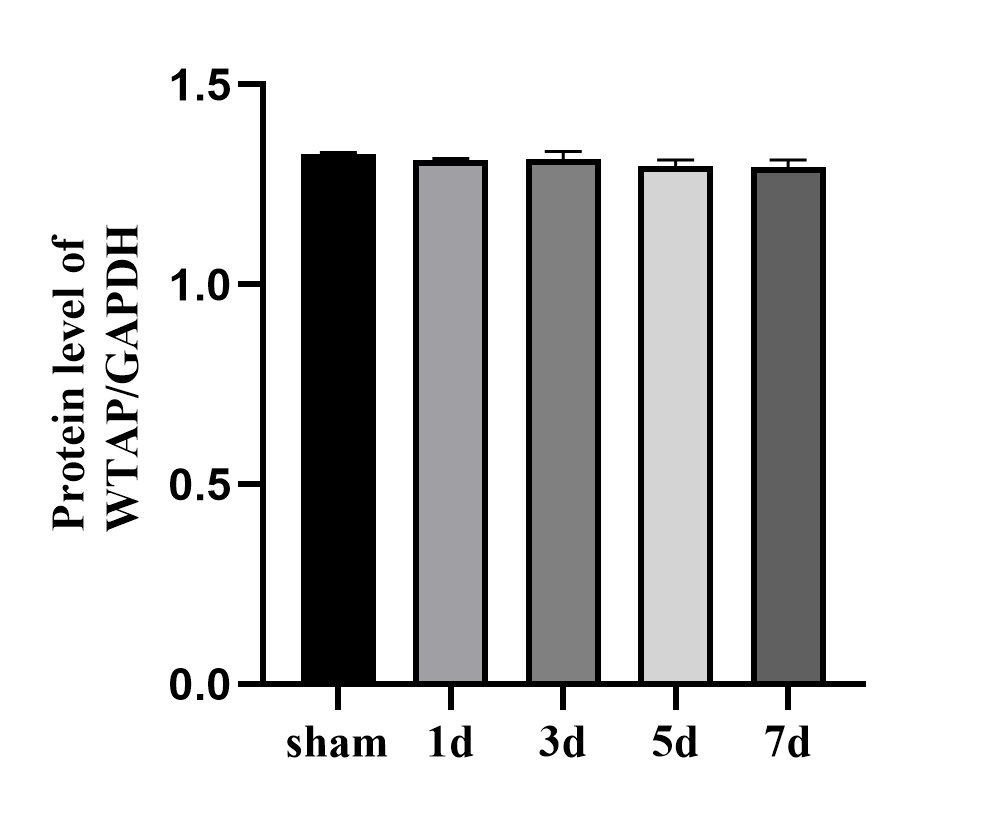


Supplementary Fig.3

A-F,The methyltransferases, METTL14 and WTAP, and demethylase, ALKBH5 showed no differences among all the groups in either mRNA or protein levels. GAPDH in Fig. 1D and S3B are the same. GAPDH in Fig. 1F and S3F are the same. Data are presented as mean ± SD. P > 0.5 versus sham group. PVN, paraventricular nucleus; MI, myocardial infarction; SD, standard deviation.
